# Correspondence

**Published:** 1888-07

**Authors:** N. E. Brill


### CORRESPONDENCE.

*To the Editors of the* JOURNAL OF COMPARATIVE MEDICINE AND SURGERY:

GENTLEMEN.—It was with great hesitation that I concluded to wield the pen once more *in re* the Pasteur question, to answer the defense of that scientist by W. H. Pendry, D.V.S., which appeared in the last number of your journal.

That M. Pasteur "is a marked man," in the words of Dr. Pendry, there can be no doubt, so well marked that his grandiloquent promises are more generally accepted for what they are worth. In fact. promises are made to do away with almost every evil that affects an agricultural community. The phylloxera was to be eradicated, anthrax was to be a thing of the past, the pelvina was to be destroyed, and finally, the rabbit pest of Australia was to yield to the strength of his all-mighty genius. I leave it to the world to judge how well he has succeeded in accomplishing his tasks.

Hence it is that "even," to quote the language of Dr. Pendry, "supposing all his theories and results in regard to *rabies* * do not satisfactorily explain themselves to a sceptical opposition," his theories and results in regard to *every other question which he has investigated do not explain themselves to rigid scientific investigation and tests.*

Perhaps it is something quite beyond dispute, "that Pasteur's name will live long after those of his enemies have been forgotten." It seems to me that I have read somewhere in history of the names of

* Italics mine.

Balsamo, otherwise known as Cagliostro, and Mesmer; yes, I think people speak of them to-day. Surely their names have lived quite long. Yet who remembers the names of Baillie and the other *savans* who composed the commission instituted by the French Government to analyze the claims of that last-named fraud, charlatan and impostor? The commission was composed of some of the most eminent scientists of the latter half of the last century, and yet their well-earned reputation scarcely outlived their lives.

To have recourse again to Dr. Pendry's words: "Looking at the results of Pasteur's labors in a fair light, we are obliged to give him the credit of having removed from the public mind the idea that there was no possible remedy for hydrophobia—a phase of his good work that strongly presented itself to my mind the morning I saw nearly seventy inoculated in his laboratory, every one of whom seemed to believe that they had been rescued from a certain death. Perhaps this belief was their salvation, apart from any result of the inoculation; but what of that, is not a rescue to such an impression a blessing, irrespective of how it is accomplished, and does not the rescuer deserve some gratitude?" We are perfectly content to allow Pasteur's claims to rest upon this statement of Dr. Pendry's, and to henceforth ascribe all of his successes to his ability as a "faith-curist."

I should here have done with Dr. Pendry were it not for the fact that he, wittingly or unwittingly, misstated my words. There is not a single sentence in my article which reads that the Belgian government "threw the matter over," as Dr. Pendry asserts; in fact, the paper quoted the language of the report of the Belgian Commission, which was that "Pasteur's method is not as yet sufficiently established," etc. Neither did I say that Mr. Victor Horsley "was a committee of one who went to Paris," but I gave the names of the individuals who composed that committee, pointing out at the same time the discrepancy between the statements of the *British Medical Journal* and of the London *Lancet*.

Unfortunately for Pasteur's cause, Dr. Pendry falls into the same error as the British Hydrophobia Commission, when he makes use of statistics. He quotes the opinion of the Commission that only "five per cent. may be safely taken to represent the average number of cases of hydrophobia following the bites of rabid dogs." How can he or the Commission reconcile this statement with another statement of the same Commission in the same report, "that of 233 persons bitten by animals in which rabies was proved, only four died; without inoculation forty (40) would have died"? I ask of Dr. Pendry and the eminent Commission: How much is five per cent. of 233? If by any possible accurate mathematical calculation they can make it 40, I shall be willing to become a convert to their opinion.

N. E. Brill, M.D.

